# Inhibition of allogeneic inflammatory responses by the Ribonucleotide Reductase Inhibitors, Didox and Trimidox

**DOI:** 10.1186/1476-9255-7-43

**Published:** 2010-08-18

**Authors:** Mohammed S Inayat, Ismail S El-Amouri, Mohammad Bani-Ahmad, Howard L Elford, Vincent S Gallicchio, Oliver R Oakley

**Affiliations:** 1 Department of Clinical Sciences, University of Kentucky, Lexington, KY 40536, USA; 2 Molecules for Health, Inc., Richmond, VA 23219, USA; 3 Departments of Biological Sciences and Public Health Sciences, Clemson University, Clemson, SC 29634, USA

## Abstract

**Background:**

Graft-versus-host disease is the single most important obstacle facing successful allogeneic stem cell transplantation (SCT). Even with current immunosuppressive therapies, morbidity and mortality rates are high. Current therapies including cyclosporine A (CyA) and related compounds target IL-2 signaling. However, although these compounds offer great benefit, they are also associated with multiple toxicities. Therefore, new compounds with a greater efficacy and reduced toxicity are needed to enable us to overcome this hurdle.

**Methods:**

The allogeneic mixed lymphocyte reaction (MLR) is a unique *ex vivo *method to study a drug's action on the initial events resulting in T-cell activation and proliferation, synonymous to the initial stages of tissue and organ destruction by T-cell responses in organ rejection and Graft-versus-host disease. Using this approach, we examined the effectiveness of two ribonucleotide reductase inhibitors (RRI), Didox and Trimidox, to inhibit T-cell activation and proliferation.

**Results:**

The compounds caused a marked reduction in the proliferative responses of T-cells, which is also accompanied by decreased secretion of cytokines IL-6, IFN-γ, TNF-α, IL-2, IL-13, IL-10 and IL-4.

**Conclusions:**

In conclusion, these data provide critical information to justify further investigation into the potential use of these compounds post allogeneic bone marrow transplantation to alleviate graft-versus-host disease thereby achieving better outcomes.

## Introduction

Graft-versus-host disease remains one the most frequent causes of morbidity in bone marrow transplantation. Current therapies address one of the six main immunosuppressive strategies in organ transplantation: proliferation, depletion, cytokines, costimulation, ischemia-reperfusion injury, and tolerance [1]. Many of these therapies are only successful in reducing acute organ rejection and do nothing for the long term survival of the graft, whilst others are associated with non-favorable side effects. The adverse effects of current treatments include hypertension, osteoporosis, hyperglycemia (steroids); hepatic dysfunction, thrombocytopenia, marrow suppression (azathioprine); limb paralysis and convulsion (cyclosporine). Therefore, the search continues for new therapeutic modalities that enable the long term survival of grafted tissue within the host with minimal side effects. To achieve this objective has led to the alternative therapeutic approach targeting key enzymes that control cell proliferation such as ribonucleotide reductase. The rate limiting step in DNA synthesis is the production of deoxynucleoside triphosphates (dNTPs) catalyzed by ribonucleotide reductase. Inhibition of ribonucleotide reductase results in reduced DNA synthesis and cell cycle arrest [2]. This has made ribonucleotide reductase inhibitors potentially attractive clinical agents for the treatment of numerous conditions characterized by excessive cell proliferation or inappropriate immune activation such as myeloproliferative disorders [3,4], psoriasis [5], sickle cell anemia [6,7], and HIV [8].

Didox and Trimidox are polyhydroxyphenyl hydroxamic acid derivatives that are more potent inhibitors of ribonucleotide reductase than the current clinical compound, hydroxyurea (HU), which targets ribonucleotide reductase [9,10]. They have been evaluated in several animal models to compare their actions to that of HU. These studies evaluate their use in animal models of HIV [9], sickle cell disease [11], and several malignancies [12] and have shown that these compounds have greater therapeutic effectiveness and lower toxicity than HU. Given the potent efficacy and low toxicity of Didox and Trimidox in animal models, and the potential utility of ribonucleotide reductase inhibitors as cytostatic agents that may influence immune cell activation, we investigated the anti-inflammatory ability of Didox and Trimidox as a therapeutic approach to improve transplant success. Our findings clearly demonstrate that these compounds inhibit both T-cell proliferation and cytokine production following anti-CD3ε stimulation as well as in allogeneic mixed lymphocyte reactions. Not only does this have implications for monotherapy, but it has been previously shown that ribonucleotide reductase inhibitors, specifically HU are able to potentiate other drugs in a combination drug therapy [13]. The studies reported here should promote further examination into the use of Didox and Trimidox as potentiators of current therapies, thereby reducing the required dose level and associated side effects to achieve similar efficacy.

## Materials and methods

### Drug Treatment

Didox and Trimidox were synthesized and kindly provided by Dr Howard Elford, Molecules for Health (Richmond, VA). All of the compounds were dissolved in 0.9% sterile saline solution then filtered through a 0.45 μm syringe top filter and stored at 4°C in the dark for a maximum of 1 week.

### Mice

Female C57BL6, BALB/c mice aged 6-8 weeks were purchased from Harlan (Indianapolis, ID) and B10.D2 mice were obtained from The Jackson Laboratories (Bar Harbor, ME). They were housed in micro-isolator cages in temperature and humidity controlled environment and were given Purina Lab Chow and water *ad libitum*. Mice were quarantined for one week post arrival as per University of Kentucky Division of Lab Animal Research (DLAR) Standard Operating Procedures (SOP). All protocols and procedures used were approved by the University of Kentucky Institutional Animal Care and Use Committee (IACUC) prior to initiation of research.

### Cell lines

CCL-1972 mouse embryonic fibroblast (MEF) cells were obtained from the American Type Tissue Culture Collection (ATCC: Manassas, VA). They were propagated in T75 canted neck culture flasks in Dulbecco's Modified Eagles Medium (DMEM: Gibco BRL, Grand Island, N.Y) supplemented with 10% fetal bovine serum (Sigma Chemical Co, St. Louis, USA) and 1% Penicillin/Streptomycin (Gibco BRL. 10,000 units per ml penicillin +10,000 mg/ml streptomycin sulphate in 0.85% saline), and maintained in an incubator (Queue: Cell culture incubator) at 37°C and 5% carbon dioxide humidified atmosphere.

### T-cell Isolation

Mice were killed and the spleens were aseptically excised and placed into a Petri dish containing 3 ml of 2 mg/ml Collagenase I (Gibco), cut into small pieces and incubated for 60 minutes at 37°C. The whole spleen suspension was then gently pushed through a 70 μm nylon filter (Falcon, BD, Franklin Lakes, NJ). The filtrate was washed twice in 15 ml of HBSS (Gibco), then resuspended in 400 μl of degassed MACS buffer (PBS pH 7.2, 2 mM EDTA and 0.5% BSA) per spleen. T-cells were separated using MACS LS separation columns as per manufacturer's instructions. Magnetic Labeling: (Pan T-Cell Isolation Kit, Miltenyi Biotec Inc, Auburn, CA) 100 μl of Biotin-antibody cocktail was added per spleen, mixed and incubated for 10 minutes at 4°C. Then, 300 μl of buffer and 200 μl of anti-biotin Microbeads were added and incubated for a further 15 minutes at 4°C. The cells were then washed in 5 ml buffer at 1200 RPM, 4°C for 10 minutes. Finally, the pellet was resuspended in 1 ml of buffer. Magnetic Separation: The LS column was prepared by pre-rinsing with 3 ml of buffer. The labeled suspension was then gently added to the column and the unlabelled effluent collected followed by 4 washes of 3 ml each to increase T-cell yield.

### T-Cell proliferation assay

Cell proliferation and viability were determined using WST-1 colorimetric assay system which is based on the cleavage of tetrazolium salt by mitochondrial dehydrogenase in viable cells (Roche Applied Sciences, Penzberg, Germany). A concentration of 5×10^4 ^cells per well was added to a 96 well flat bottom plate (Costar, Corning, NY) pre-coated with immobilized anti-CD3ε (with or without drug treatment) and incubated at 37°C and 5% CO_2_. Proliferation was determined as per the manufacturer's instructions at 24, 48, 72 and 96 hours post treatment.

### Cytokine Analysis

T-cells (1×10^5 ^per well) were incubated at 37°C and 5% CO_2 _in 96-well plates (Costar, Corning, NY) coated with anti-CD3ε monoclonal antibodies (eBioscience, San Diego, CA). Culture supernatants were taken at 24 and 48 hour time points; the supernatants were spun free of cells and aliquots were frozen at -80°C. Levels of cytokine secretion (IL-2, IL-4, IL-6, IL-10, IL-12p70, IL-13, IFN-γ and TNF-α) were analyzed with the Searchlight multiplex assay system (Pierce Biotechnology Inc, Woburn, MA). Briefly, Custom 96 well culture plates (Costar) were manufactured which contained target capture antibodies as indicated above. 50 μl of the supernatant was then added to each well for 1 hour, followed by three washes and the addition of biotinylated secondary antibodies for 30 minutes. The wells were then washed again and streptavidin-horseradish peroxidase (SA-HRP) conjugate was added followed by the addition of SuperSignal^® ^ELISA Femto chemiluminescent substrate. The luminescence was then detected and analyzed using a cooled charge-coupled device imager (Pierce Biotechnology Inc).

### Mixed Lymphocyte Reaction

T-cell depleted stimulator cells were obtained from splenocytes from BALB/c mice by magnetic bead purification (Miltenyi Biotec). The cells were collected in RPMI 1640 growth media (Gibco) supplemented with 10% fetal bovine serum (Sigma), 50 μM 2-mercaptoethanol (Sigma) and 1% penicillin streptomycin (Gibco) and irradiated with 3000 rads of γ-radiation form a Cs-137 source.

Responder T-cells were isolated as previously described (Miltenyi Biotec) from C57BL6 (Major antigen mismatch) mice and B10.D2 (Minor antigen mismatch) mouse strains. A total of 2 × 10^5 ^responder cells were combined with 4 × 10^5 ^stimulator cells per well in 96 well flat bottom plates (Costar) and incubated at 37°C and 5% CO_2_. T-cell culture media, RPMI 1640 (Gibco) containing 10% fetal bovine serum (Sigma), 50 μM 2-mercaptoethanol (Sigma) and 1% penicillin streptomycin (Gibco) was supplemented with either Didox or Trimidox from 25 μM-100 μM or PBS and the MLRs were analyzed in triplicate on day 6.

### Statistical analysis

When applicable, results were subjected to statistical analysis. Data were analyzed and plotted using SigmaPlot version 10.0 (Systat software Inc., Chicago, IL). On graphs, error bars represent one standard error (± SE) around the average of data per group. To determine the statistical significance between groups, ANOVA was performed followed by post-hoc analysis using Bonferroni method. Data that failed normality testing was normalized using log transformation. *p *values < 0.05 were considered significant.

## Results

### Didox and Trimidox inhibit T-cell proliferation in CD3ε stimulated cultures

**Figure 1:**
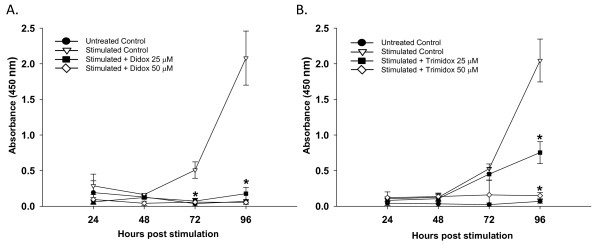
**Inhibition of proliferation of T-cell obtained from B10.D2 mice spleens**. Briefly, 5 × 10^4 ^T-cells per well were purified and seeded in 96 well sterile plates pre-coated with PBS or anti CD3ε antibodies (5 μg/ml). Either PBS or several concentrations of Didox or Trimidox (25 μM and 50 μM) was added to RPMI 1640 growth media supplemented with 10% fetal bovine serum, 50 μM 2-Mercaptoethanol and 1% penicillin streptomycin. The plates were then incubated at 37°C and 5% CO_2 _for 24, 48, 72 or 96 hours, after which spectrophotometric quantification of cell growth and viability was determined. The results shown represent data obtained in triplicate from two independent experiments. **(A) **Treated with Didox **(B) **Treated with Trimidox. Values shown (mean ± SD) represent data obtained in triplicate from two independent experiments. * indicate a significant difference compared to anti CD3ε stimulated. (*p *< 0.05, ANOVA + the Bonferroni test).

**Figure 2:**
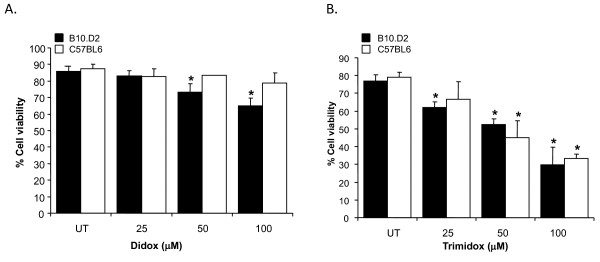
**Cellular toxicity of Didox and Trimidox in T-cells from C57BL6 and B10.D2 mouse strains**. Briefly, 1 × 10^5 ^cells per well were seeded in 96 well culture plates in RPMI 1640 supplemented with 10% fetal bovine serum and 1% penicillin streptomycin and 2-mercaptoethanol, containing PBS or concentrations of Didox or Trimidox from 25 μM- 100 μM. The cells were then incubated at 37°C and 5% CO_2 _for 4 days. After this, percentage of viable cells of **(A) **Didox and **(B) **Trimidox was determined for each drug dose. * indicates a significant difference compared to PBS treated (UT). (*p *< 0.05, ANOVA + the Bonferroni test; n = 3).

To determine the influence of Didox and Trimidox on T-cell proliferation, we first studied the effects of the compounds on T-cell proliferation in response to CD3ε stimulation. Untreated control cultures did not show detectable proliferation throughout the experiments. Following stimulation, proliferation was detected in stimulated control cultures at 48 hours. The control cultures continued to proliferate rapidly until the last time point assayed at 96 hours post stimulation (Figure 1). Didox (Figure 1A) treatment at the lowest concentration (25 μM) reduced T-cell proliferation by approximately 90% at 96 hours post stimulation, and proliferation was undetectable when treated with 50 μM. Trimidox (Figure 2B) at 25 μM inhibited proliferation by approximately 65% compared to stimulated control at 96 hours post stimulation and completely blocked proliferation at 50 μM concentration.

### Suppression of T-cell proliferation by Didox and Trimidox is not due to the cytotoxic effects of the treatment

To determine if the lack of proliferation was a result of the toxicity of the compounds, we studied the effects of drug treatment on cell viability. In the proliferation studies, we observed that 50 μM of Didox or Trimidox was sufficient to completely block T-cell proliferation (Figure 1). Figure 2A. shows cell viability of B10.D2 and C57BL6 T-cells in response to Didox treatment. The results indicate that C57BL6 cells are more tolerant to the treatment than B10.D2 cells. Even so, Didox treatment only reduced cell viability in the B10.D2 cells by 20% at 100 μM. The C57BL6 cells were more tolerant to the compound and had a minimal (5%) reduction in cell viability at the highest concentration used (100 μM). Figure 2B shows cell viability in response to Trimidox treatment. We observed an approximate 15% reduction in cell viability at the minimal effective dose (25 μM). Cell viability in both B10.D2 and C57BL6 was decreased to less than 50% when cells were exposed to 100 μM concentrations.

### Suppression of anti CD3ε induced cytokine production in T-cell cultures by Didox and Trimidox

**Figure 3:**
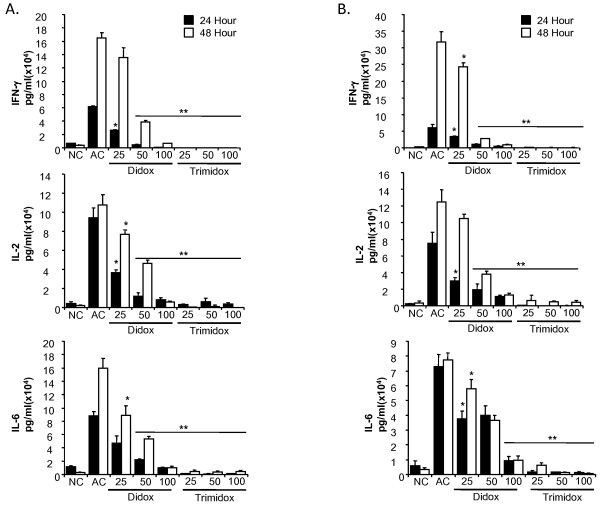
**Inhibition of TH1 cytokine secretion by Didox and Trimidox**. Briefly, 2 × 10^5 ^T-cells per well were purified and seeded in 96 well sterile plates pre-coated with PBS or anti CD3ε antibodies (5 μg/ml). Either PBS or several concentrations of Didox or Trimidox (25 μM- 100 μM) was added to RPMI 1640 growth media supplemented with 10% fetal bovine serum, 2-mercaptoethanol (50 μM) and 1% penicillin streptomycin. The plates were then incubated at 37°C and 5% CO_2 _for 24 or 48 hours, after which the levels in pg/ml of IFN-γ, IL-2 and IL-6 were determined using the Searchlight multiplex assay system. **(A) **B10.D2 mice; **(B) **C57BL6 mice. NC: Normal control, AC: Activated control. The values shown (mean ± SD) represent data obtained in triplicate from two independent experiments. * *p *< 0.05, ** *p *< 0.001 indicates a significant difference compared to activated control (AC), ANOVA + the Bonferroni test; n = 3.

We next studied the effects of Didox and Trimidox on cytokine production. As mentioned previously, many current therapies target cytokine signaling or production, in particular the CyA derivatives inhibit signaling through IL-2. T-cell cultures stimulated with anti-CD3ε were tested for cytokine activity using the Searchlight multiplex assay at 24 and 48 hours post stimulation. We tested both B10.D2 (Figure 3A) and C57BL6 (Figure 3B) cells independently. As expected, the unstimulated (NC) (no anti-CD3ε treatment) cells showed minimal cytokine production. However, in as little as 24 hours, both C57BL6 and B10.D2 cells responded to anti-CD3ε (AC) by increasing secretion of IFN-γ, IL-2 and IL-6. In contrast, cells treated simultaneously with Didox and anti-CD3ε demonstrated a dose response inhibition of cytokine production at 24 or 48 hours post stimulation compared to the activated controls (AC) (*p *< 0.02).

We observed an even greater inhibition of cytokine production by Trimidox following anti-CD3ε stimulation of T-cell cultures in both B10.D2 and C57BL6 mice. The production of IFN-γ was below detectable levels in both cell types at either 24 or 48 hours. The production of IL-2 in both cell types was comparable to that of unstimulated cells of the same strain; this pattern was also true for IL-6 production.

**Figure 4:**
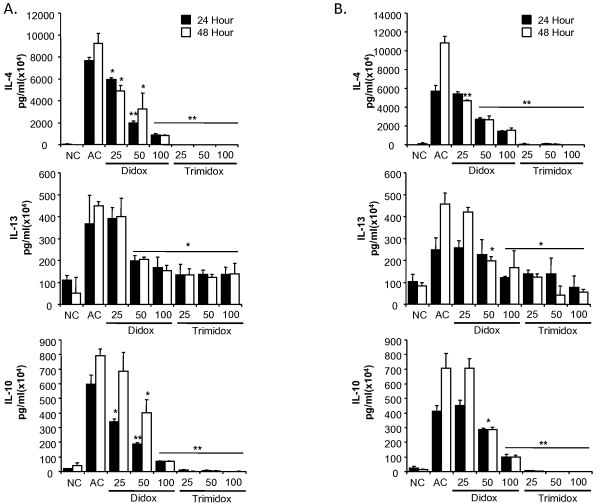
**Inhibition of TH2 cytokine secretion by Didox and Trimidox**. The levels in pg/ml of IL-4, IL-13 and IL-10 (*p < 0.05, ** *p *< 0.001) were determined using the Searchlight procedure. **(A) **B10.D2 mice; **(B) **C57BL6 mice. NC: Normal control, AC: Activated control. The values shown (mean ± SD) represent data obtained in triplicate from two independent experiments. * *p *< 0.05, ** *p *< 0.001 indicates a significant difference compared to activated control (AC), ANOVA + the Bonferroni test; n = 3.

We also examined the production of several Th2 type cytokines IL-4, IL-13 and IL-10 (Figure 4). Normal control cell supernatants contained minimal levels of IL-4, IL-13 and IL-10. Following stimulation with anti-CD3ε, cultures rapidly produced increased levels of Th2 type cytokines at 24 hours post stimulation that further increased at 48 hours post stimulation. Treatment with Trimidox, even at the lowest dose (25 μM), reduced the levels of IL-4 and IL-10 to the lower detection limits. Interestingly, Trimidox treatment in either B10.D2 or C57BL6 mice reduced levels of IL-13 to levels comparable to normal controls (NC). Didox treatment demonstrated a dose dependant decrease in cytokine levels for IL-4, IL-10 and IL-13.

### The effects of Didox and Trimidox on Allogeneic MLR

**Figure 5:**
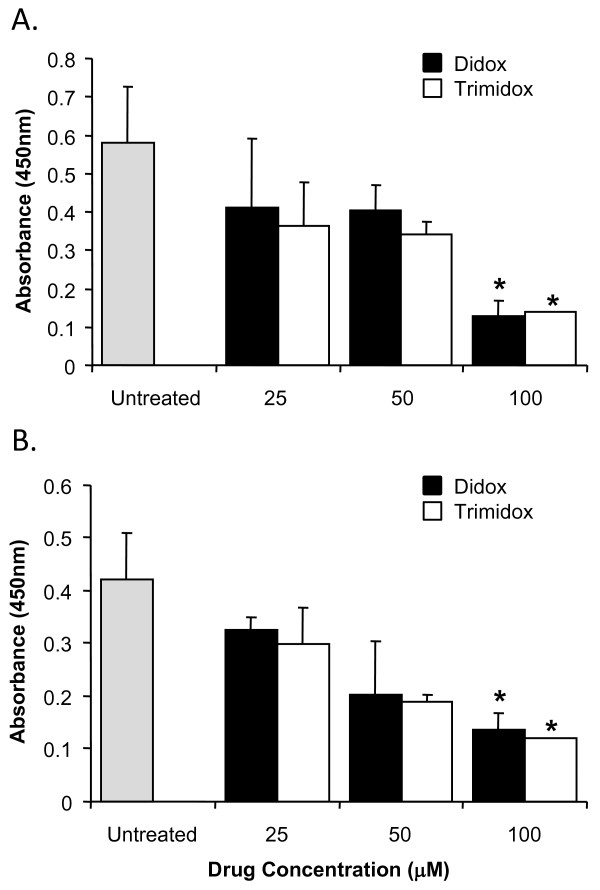
**Inhibition of T-cell proliferation responses in MLRs by Didox and Trimidox**. (A) Major antigen mismatch: Briefly, 2 × 10^5 ^T-cells were purified from spleens C57BL6 mice (responder cells) were mixed with 4 × 10^5 ^irradiated non T-cells (stimulator cells) obtained from BALB/c mice (exposed to 3000 rads of γ-radiation). **(B) **Minor antigen mismatch: Briefly, 2 × 10^5 ^T-cells were purified from spleens B10.D2 mice (responder cells) which were mixed with 4 × 10^5 ^non T-cells (stimulator cells) obtained from BALB/c and pre-exposed to 3000 rads of γ-radiation. Either PBS (untreated) or several concentrations of Didox or Trimidox (25 μM- 100 μM) was added to RPMI 1640 growth media supplemented with 10% fetal bovine serum, 50 μM 2-Mercaptoethanol and 1% penicillin streptomycin. The plates were then incubated at 37°C and 5% CO_2 _for 6 days, after which spectrophotometric quantification of cell growth and proliferation was determined. The results shown represent data obtained in triplicate from two independent experiments. Values shown represent the mean ± SD obtained in triplicate from two independent experiments. * indicates a significant difference compared to PBS treated (Untreated). (*p *< 0.05, ANOVA + the Bonferroni test).

The following experiments were performed to analyze the effects of Didox and Trimidox in the complex interaction of allo-recognition and activation of responder T-cells to both major (Figure 5A) and minor (Figure 5B) mismatched antigens. In the first set of experiments, C57BL6 lymphocytes were stimulated with irradiated BALB/c stimulators. Didox and Trimidox were added at 25 μM, 50 μM and 100 μM following initiation of the cultures. As shown in Figure 5A., untreated cultures demonstrated an increase in T-cell proliferation. The addition of Didox or Trimidox at either 25 μM or 50 μM caused 40-45% inhibition of proliferation. The addition of 100 μM of Didox or Trimidox caused a 75-80% inhibition of proliferative responses. The second set of experiments used T-cells from B10.D2 mice as the responders and irradiated non-T-cells from BALB/c mice as the stimulators. Figure 5B. shows the inhibition of proliferative responses by both Didox and Trimidox in a dose dependant manner. Here we show a 20-25% inhibition by both drugs at 25 μM, a 45-50% inhibition by both drugs at 50 μM, and a 70-75% inhibition by both drugs at 100 μM.

### Didox and Trimidox inhibit cytokine production during the MLR

**Figure 6:**
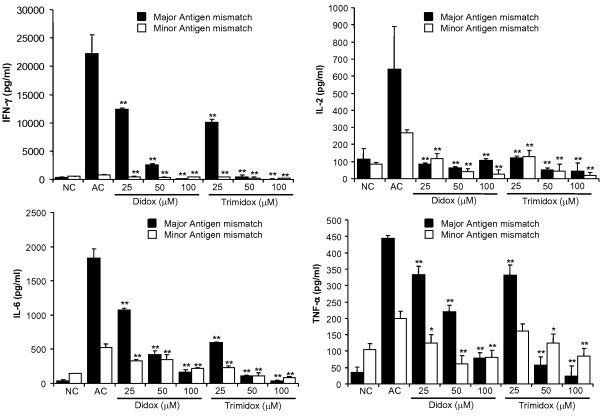
**Inhibition of IFN-γ, IL-2, IL-6 and TNF-α cytokine release from MLRs by Didox and Trimidox**. Major antigen mismatch (filled square): Briefly, 2 × 10^5 ^T-cells were purified from spleens of C57BL6 mice (responder cells) which were mixed with 4 × 10^5 ^non T-cells (stimulator cells) obtained from BALB/c mice and exposed to 3000 rads of γ-radiation. Minor antigen mismatch (open square): Briefly, 2 × 10^5 ^T-cells were purified from the spleens of B10.D2 mice (responder cells) which were mixed with 4 × 10^5 ^non T-cells (stimulator cells) obtained from BALB/c mice and previously exposed to 3000 rads of γ-radiation. Either PBS or several concentrations of Didox or Trimidox (25 μM- 100 μM) was added to RPMI 1640 growth media supplemented with 10% fetal bovine serum, 2-mercaptoethanol (50 μM)and 1% penicillin streptomycin. The plates were then incubated at 37C and 5% CO_2 _for 6 days, after which the levels of IFN-γ, IL-2 and IL-6 were determined using the Searchlight multiplex assay system. NC: Normal control, AC: Activated control. The results shown represent data obtained in triplicate from two independent experiments (n = 3). * *p *< 0.05, ** *p *< 0.001 indicates a significant difference compared to activated control (AC), ANOVA + the Bonferroni test.

In an extension of the anti-CD3ε studies in which we observed an inhibition of cytokine production, we performed similar Searchlight multiplex analysis on 6 day culture supernatants from either major or minor antigen mismatched MLRs. Figure 6 shows the results for IFN-γ, TNF-α, IL-2, and IL-6 cytokine levels from cell culture supernatants in response to minor and major antigen stimulation. The base line production of cytokines is represented in each graph by the normal control (NC), unstimulated responder cells and untreated activated control (AC). The minor MHC antigen stimulated cultures showed only a minimal increase in cytokine production, ranging from only a 25% increase in IFN-γ to a 250% increase in IL-2. In sharp contrast, however, the major antigen stimulation resulted in a 78.4 fold increase in IFN-γ expression when compared to the normal control group (Figure 6). These data clearly demonstrate the inflammatory effect of major MHC mismatched antigens to stimulate a potent cytokine response for all four cytokines shown. A similar trend in the secretion of IL-2 was observed, with only a 2-fold increase in response to minor antigens and > 6 fold increase with the major MLR (Figure 6). An increase in IL-6 production was seen in both the major MLR and minor MLR when compared to the normal controls.

Treatment with Didox or Trimidox was able to inhibit MLR induced cytokine production in a dose dependent manner. Trimidox inhibited cytokine production to background levels at a dose of 25 μM for IL-2, 50 μM for IFN-γ, 100 μM for TNF-α and 100 μM for IL-6. In contrast, a higher dose of Didox (100 μM) was needed to inhibit IFN-γ and IL-2 production to levels comparable to NC.

**Figure 7:**
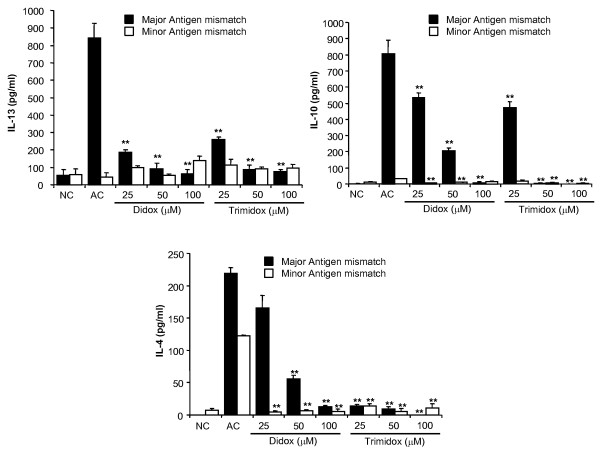
**Inhibition of IL-13, IL-10 and IL-4 cytokine release from MLRs by Didox and Trimidox**. The levels of IL-13, IL-10 and IL-4 were determined using the Searchlight multiplex assay system from minor (open square) or major (filled square) mixed lymphocyte reactions. NC: Normal control, AC: Activated control. The results shown represent data obtained in triplicate from two independent experiments (n = 3-6). (*p < 0.05, ** *p *< 0.001 indicates a significant difference compared to activated control (AC), ANOVA + the Bonferroni test.

As shown in Figure 7, major antigen mismatched MLRs resulted in a rapid upregulation of the Th2 derived cytokines, IL-13, IL-4 and IL-10. In the minor antigen MLRs, a significant increase was only detected in the production of IL-4, which was quenched by the lowest dose (25 μM) of either Didox or Trimidox. NC (unstimulated responder cells) had minimal levels of cytokine production. Didox inhibited cytokine production in a dose dependant manner for IL-13, IL-10 and IL-4, with the 100 μM dose of Didox inhibiting cytokine levels comparable to that in NC. Trimidox inhibited cytokine production more vigorously than Didox with a dose of 25 μM inhibiting IL-10, IL-13, and IL-4 by approximately 40%, 60% and 90% respectively. Levels of cytokines were reduced to or below that of NC by treatment with a 50 μM dose. Together, these data demonstrate the ability of the ribonucleotide reductase inhibitors, Didox and Trimidox, to act as inhibitors of inflammatory responses by both inhibiting the proliferation of T-cells and the production of cytokines.

## Discussion

In this study, we determined the effect of Didox and Trimidox on the proliferative response of T-cells. Although ribonucleotide reductase inhibitors, specifically HU, have been used extensively to synergize the actions of other compounds, such as the combination of HU with didanosine in the treatment of HIV [14,15], recent studies have identified other potential applications based on their cytostatic properties [16]. The results of this report demonstrate the ability of Didox and Trimidox to function as cytostatic compounds by inhibiting T-cell proliferation that occurs as a consequence of SCT or solid organ transplantation. In addition, the experiments presented here demonstrate that Didox and Trimidox inhibit the proliferation of not only T-cells following anti-CD3ε stimulation, but also in MLRs. Previous studies have identified T-cell proliferation, cytokine secretion, and the resulting increased expression of chemokines as crucial events following solid organ or stem cell transplant (SCT). By specifically targeting these responses, these compounds have the potential to limit graft rejection or graft-versus-host disease [17,18].

The process of graft rejection is a complicated yet well orchestrated event; the precise mechanisms are still not completely understood. It is known that T-cells alone are not sufficient for graft rejection, that cytokines are required and that rejection is driven by a Th1-type pattern of immune activation [19], and as such, a switch to a predominantly Th2-type pattern can be beneficial to graft survival at the cost of increased opportunistic infections.

Previous work has shown that IL-2, IL-6, TNF-α and IFN-γ secretion is associated with acute graft-versus-host disease [20,21], whilst increased circulatory levels of IL-6 have been associated with chronic graft-versus-host disease [22]. More recently, Mohty review the clinical significance of Th1 cytokines in both amplification of donor responses and direct cytotoxicity [23]. Hoping to alleviate these detrimental events, we demonstrate here, the ability of these compounds to inhibit the secretion of the crucial cytokines, IL-2, IL-6, TNF-α and IFN-γ thereby reducing the collateral damage caused by the direct actions of these cytokines, whilst decreasing the proliferative capacity of the T-cells. The differential inhibition of cytokines and T-cell proliferation may be an indication that the two compounds, Didox and Trimidox may actually be functioning by two distinct mechanisms. The first as an inhibitor of ribonucleotide reductase, with its effects resulting in a reduction of T-cell proliferation; the second mechanism may be acting at another level, directly reducing inflammatory cytokine levels. Previous studies show that both Didox and Trimidox directly inhibit NFκB phosphorylation at concentrations comparable to this study [24]. Therefore, the second mechanism, inhibiting cytokine levels may be a result of Didox and Trimidox inhibition of NFκB.

The concept of using drugs to inhibit T-cell proliferation is not a new one; however, a major drawback of this approach has been the concurrent inhibition in any residual anti-tumor, or graft versus leukemia (GVL) effect of lymphocytes that remain. The beneficial effects of GVL responses have been well documented [25-28]. This GVL effect is one that is most notably seen in allogeneic as opposed to autologous bone marrow transplants and is dependent on the genetic mismatch between graft and recipient. However, in order for this retained GVL effect to be effective, T-cells populations must also be able to activate and proliferate in response to antigen, whether it is allo-antigen (major or minor), tumor-antigen, tumor-associated antigen or a combination of all three.

Recent publications have demonstrated certain ribonucleotide reductase inhibitors function as virostatics [8,29] and that this effect may be an additional benefit. More recently, the immune modulatory effects of HU have been postulated. Lori *et al *described the "predator-prey" hypothesis [29] where the cytostatic effects of HU would force T-lymphocytes into becoming quiescent, thus becoming less prone to HIV infection. The overall effect is fewer numbers of infected cells and reduced viral loads and ultimately more effective control by host immune responses. Benito *et al *[30] described the anti-proliferative effects of HU on T-cells without diminishing their cellular activation.

In addition to Didox and Trimidox being considered for use as anti-proliferative agents, current research has indicated that they are also successful as anti-tumor agents. Raje, et al [31] demonstrates that Didox specifically induces a caspase-dependant cytotoxicity in multiple myeloma (MM) cells. They demonstrate that not only does Didox induce apoptosis in MM cells, but that this is accompanied by a down-regulation of several other genes including bcl-2, bclx1, and XIAP as well as a reduction in both expression and protein levels of M1 subunit of ribonucleotide reductase. They also demonstrated a myeloma-specific down-regulation of RAD 51 homologue, an active gene in DNA repair. The authors conclude that Didox acts on both DNA synthesis and repair.

In contrast to studies using HU [32], Didox and Trimidox also inhibited the production of Th2 type cytokines. These cytokines are known to suppress many pro-inflammatory cytokines and chemokines. In the transplant scenario, upregulation of these cytokines may be important in the generation of humoral responses. The differential inhibition of Th1 cytokines by HU results in a net increase in Th2 type cytokines, thus further lowering the immune response and increasing the potential for opportunistic infections. Here we show that Didox and Trimidox inhibit both Th1 and Th2 cytokines possibly leaving the Th1:Th2 balance intact although at a reduced level. The *in vivo *implications of these data are important due to many factors. The secretion of IFN-γ in the post transplant setting is important in both direct cellular damage to host tissues and also in the stimulation of cell mediated responses [33]. Increased levels of IL-6 have also been found to be a negative predictor of graft survival in numerous transplant scenarios [34,35]. The widespread effect of IL-2 on T-cell proliferation has been well documented and thus serves as a target for many of the current immunosuppressive therapies. Our data implies that Didox and Trimidox can be important tools to inhibit the proliferation of T-cells in the transplant setting by both inhibiting T-cell proliferation and reducing detrimental cytokine secretion. However, the question remains whether these treatments would impair the normal host defenses against microbial insults. Weinberg [32] suggests that the action of ribonucleotide reductase inhibitors would not impair immunological responses to opportunistic pathogens as their actions are limited to stimulated lymphocytes and that unstimulated PBMCs are unaffected.

Finally, one of the major side effects of current ribonucleotide reductase inhibitor treatment is the potential for myelotoxicity, an unfavorable side effect post transplant. These effects can be directly damaging to the bone marrow by preventing the mobilization of stems cells and thus affecting the self-renewal capacity of the bone marrow as a whole. As such, this myelotoxicity can persist for many years following cessation of treatment further impairing future treatments. However, previous work has shown the improved antiviral effects of Didox and Trimidox with a limited myelosuppressive effects [10] compared to HU, which indicates that, unlike HU, myelotoxicity would not be as problematic when using Didox or Trimidox.

To summarize, allogeneic SCT results in the up regulation of a plethora of chemokines, cytokines, and transcription factors which culminate in the homing/localization and emergence of host reactive T-cells resulting in graft-versus-host disease in the recipient [36,37]. These studies demonstrate that both Didox and Trimidox, at doses achievable *in vivo*, have anti-proliferative effects on T-cells in response to allo-antigens and downregulate crucial cytokine secretion associated with graft versus host disease. These findings indicate that Didox and Trimidox act in a multifaceted manner, and as such, would be suitable candidates for further evaluation in animal models of solid organ transplant, graft-versus-host disease and autoimmunity.

## Abbreviations

IFN-γ: interferon-gamma; MLR: mixed lymphocyte reaction; RRI: ribonucleotide reductase inhibitor; CyA: cyclosporine A; GVHD: graft-versus-host disease.

## Competing interests

HE is President and a shareholder of Molecules for Health (MFH) and thereby has a financial interest in Didox or Trimidox therapeutic potential. All other authors declare that they have no competing interests.

## Authors' contributions

MI participated in the design of the experiments, carried out the T-cell isolation, T-cell proliferation, cytokine analysis and MLR and analysis of the data. IE participated in the T-cell proliferation assays and statistical analysis. MB participated in the T-cell proliferation assays. HE supplied the compounds Trimidox and Didox and participated in the determination of the in vitro dosing. VG assisted in drafting the manuscript. OO conceived the study, directed its design and coordination and drafted the manuscript. All authors read and approved the final manuscript.
